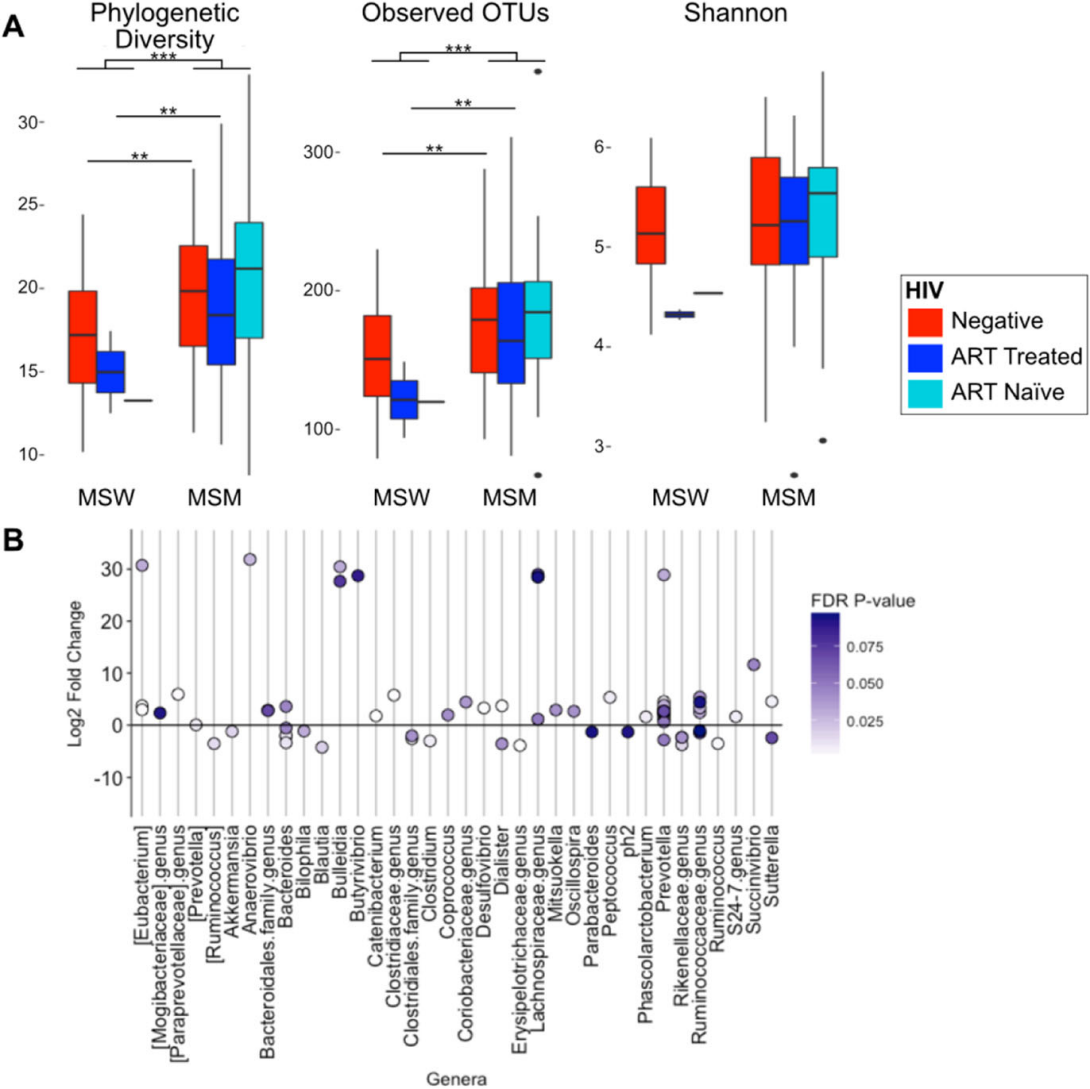# Correction to: An exploration of *Prevotella*-rich microbiomes in HIV and men who have sex with men

**DOI:** 10.1186/s40168-020-00829-6

**Published:** 2020-04-06

**Authors:** Abigail J. S. Armstrong, Michael Shaffer, Nichole M. Nusbacher, Christine Griesmer, Suzanne Fiorillo, Jennifer M. Schneider, C. Preston Neff, Sam X. Li, Andrew P. Fontenot, Thomas Campbell, Brent E. Palmer, Catherine A. Lozupone

**Affiliations:** 1 Department of Medicine, University of Colorado Denver, Anschutz Medical Campus, Aurora, CO USA; 2 Department of Immunology and Microbiology, University of Colorado Denver, Anschutz Medical Campus, Aurora, CO USA; 3 Computational Bioscience Program, University of Colorado Denver, Anschutz Medical Campus, Aurora, CO USA; 4 Department of Surgery, University of Colorado Denver, Anschutz Medical Campus, Aurora, CO USA

**Correction to: Microbiome (2018) 6:198**

**https://doi.org/10.1186/s40168-018-0580-7**

Following publication of the original article [1], the authors reported an error in Fig. 2. The original Fig. 2 has been incorrectly replaced with the Supplementary Fig. 2. The correct Fig. 2 is presented here.