# Supplementary figures and images for: An exploration of Prevotella-rich microbiomes in HIV and men who have sex with men
Source: Microbiome. 2018 Nov 5;6:198. doi: 10.1186/s40168-018-0580-7 (PMC6219090; doi:10.1186/s40168-018-0580-7)

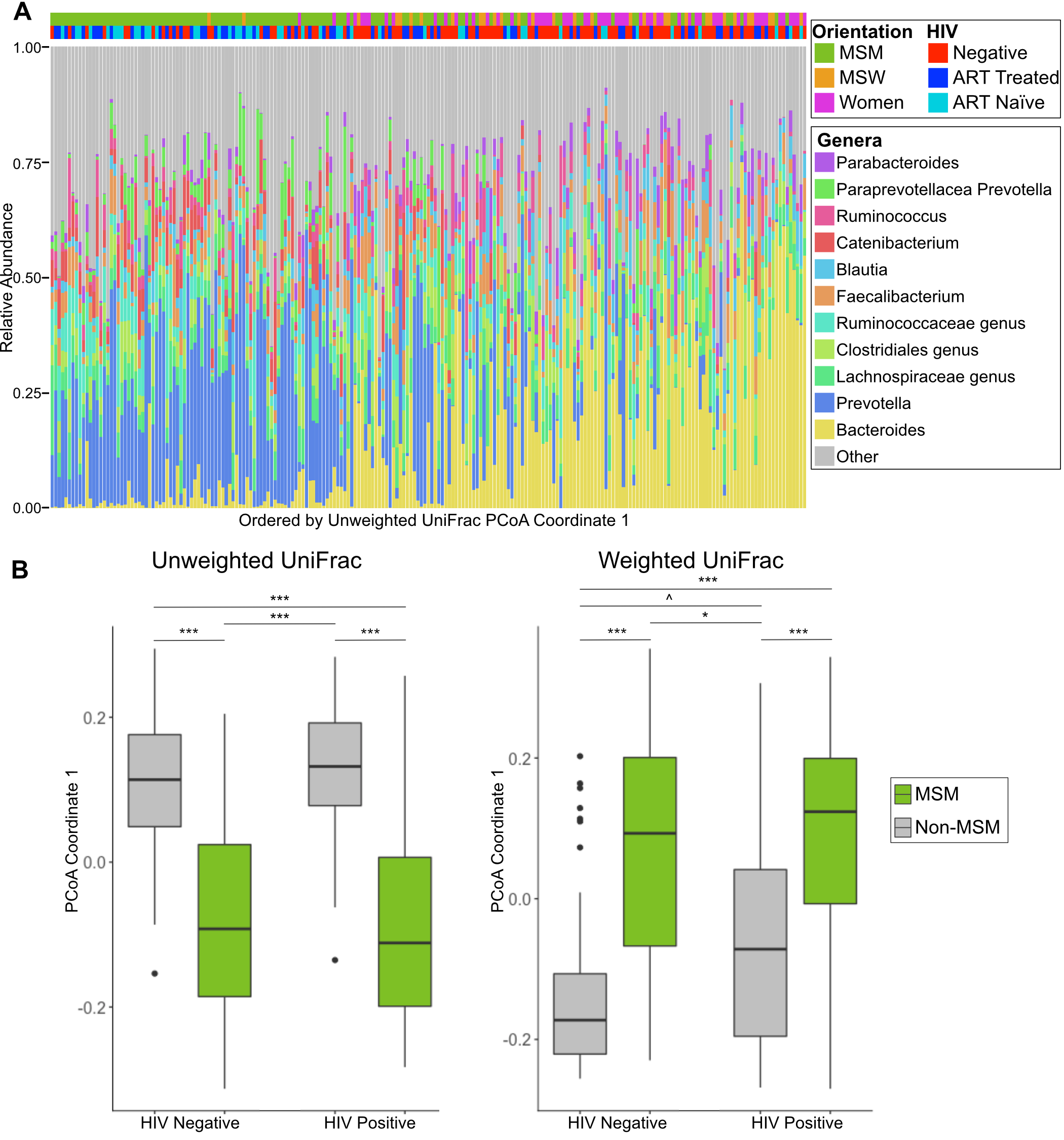

Supplement: Supplementary file 4 — Figure S1. Principal coordinate analysis for taxonomy and clustering. A. Genus level taxa bar chart order by unweighted UniFrac PCoA coordinate 1. (Bottom) Genera with mean relative less than 2% abundance are binned together into the category “Other”. Each column represents one individual. (Top) samples are marked with HIV status and orientation. Each column corresponds to the genus plot below. Samples are ordered by the coordinate on the weighted UniFrac principle coordinate 1. B. Orientation and HIV “clustering” along principal coordinate 1 in unweighted (left) and weighted (right) UniFrac. (Kruskal-Wallis test, p < 0.001; Dunn’s Post Hoc Test, FDR P-value: ^ < 0.1, * < 0.05, ** < 0.01, *** < 0.001) (PNG 294 kb) [file 40168_2018_580_MOESM4_ESM.png]

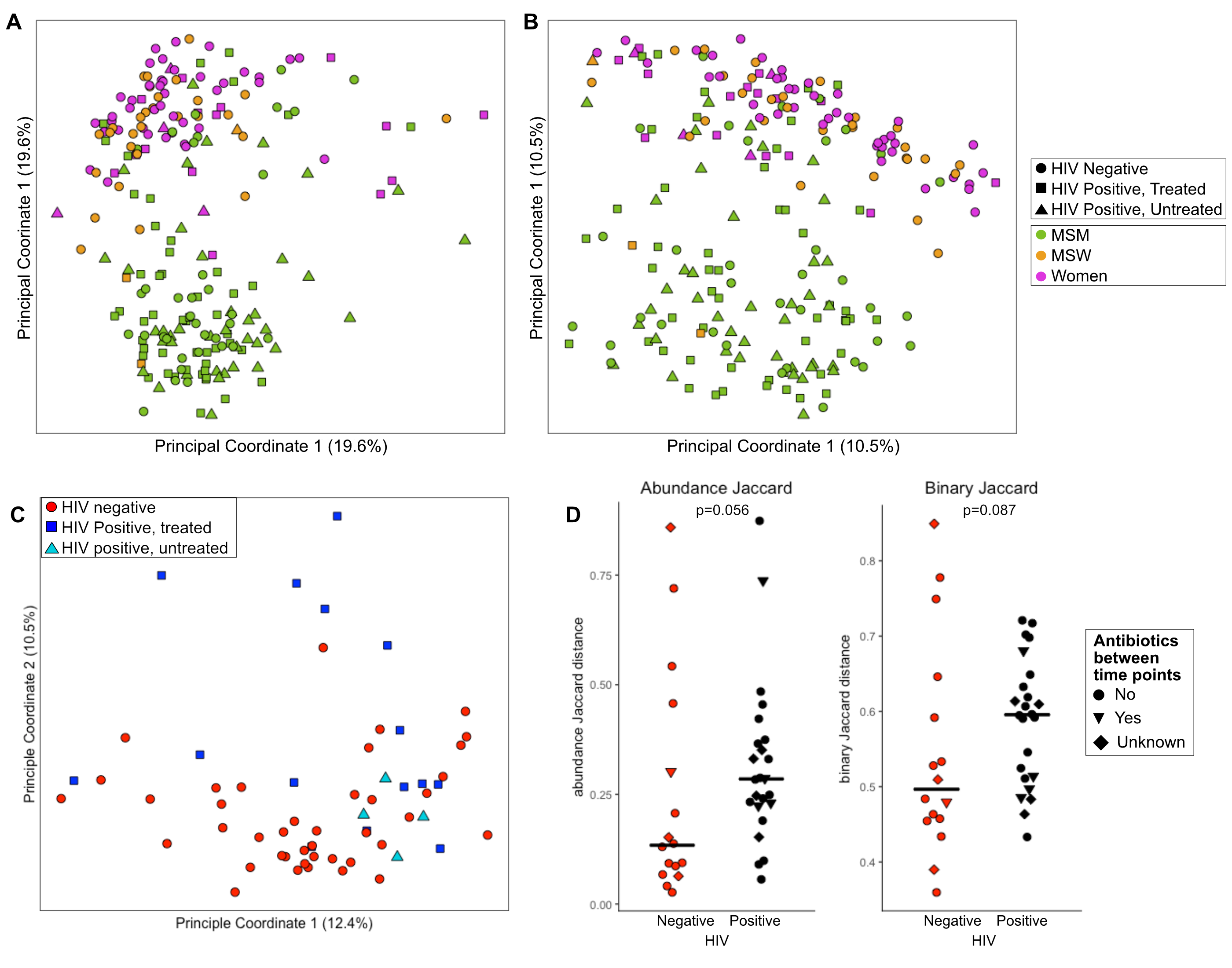

Supplement: Supplementary file 5 — Figure S2. Abundance and Binary Jaccard Analysis. A. Abundance and B. binary Jaccard with points colored by orientation and shaped by HIV status in all study samples. C. Abundance Jaccard of women only; points colored by HIV status. D. Abundance (left) and binary (right) Jaccard of longitudinal samples comparing HIV negative individuals to HIV-positive pre- and post-ART initiation (Kruskal-Wallis test). (PNG 764 kb) [file 40168_2018_580_MOESM5_ESM.png]

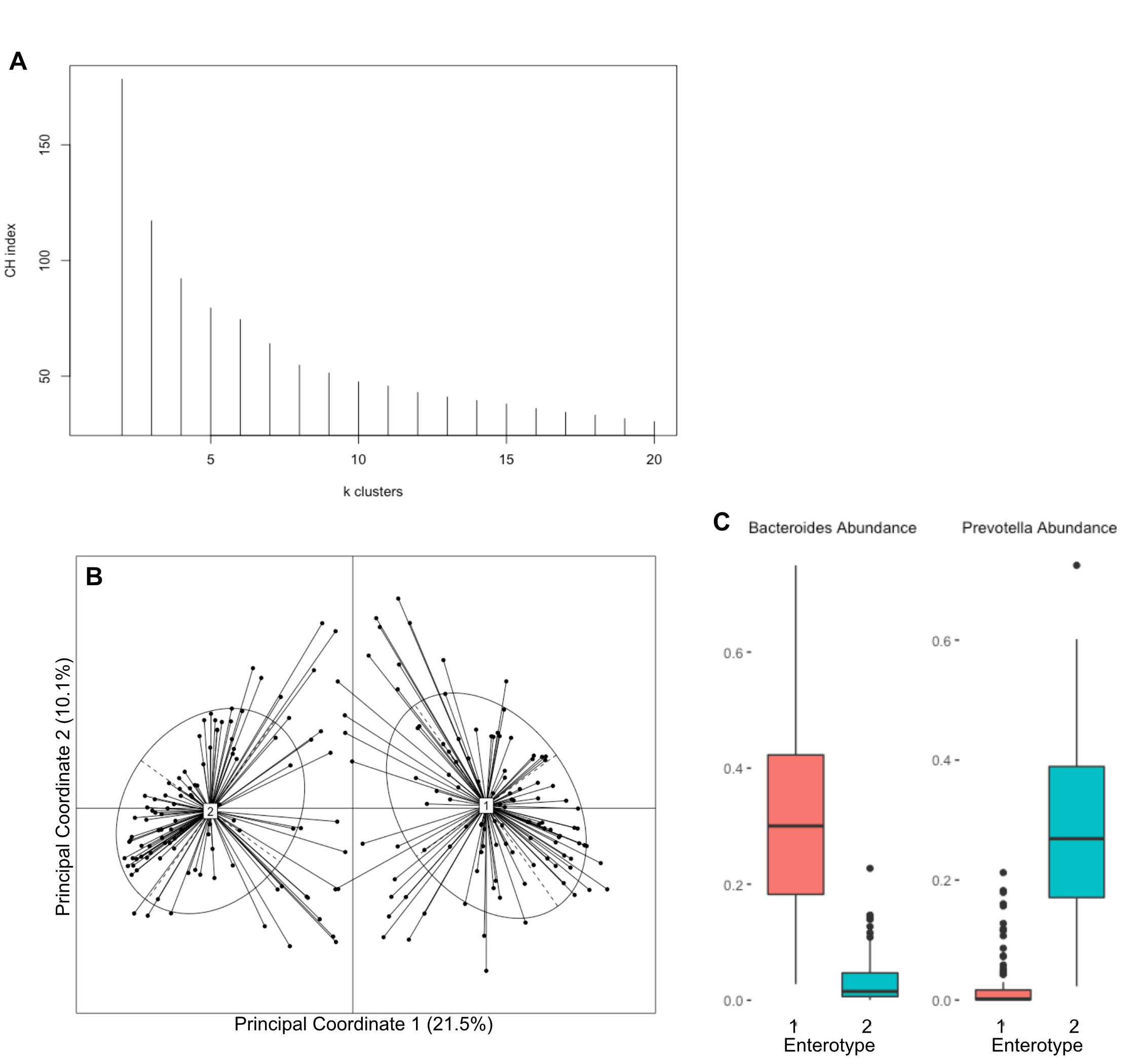

Supplement: Supplementary file 6 — Figure S3. Samples are best divided into two enterotypes, which are primarily defined by Bacteroides or Prevotella richness. A. Silhouette analysis shows that two clusters is the most efficient way to divide the data. B. PCoA of Jensen–Shannon divergence distance. The center of each enterotype/cluster is marked with a line to each member of the cluster. Centers were calculated using Partitioning around medoids (PAM). C. The relative abundance of the two most abundant taxa in the clusters, Prevotella and Bacteroides. Each enterotype is marked by a Prevotella-rich/Bacteroides-poor or Bacteroides-rich/Prevotella-poor composition. (PNG 481 kb) [file 40168_2018_580_MOESM6_ESM.png]

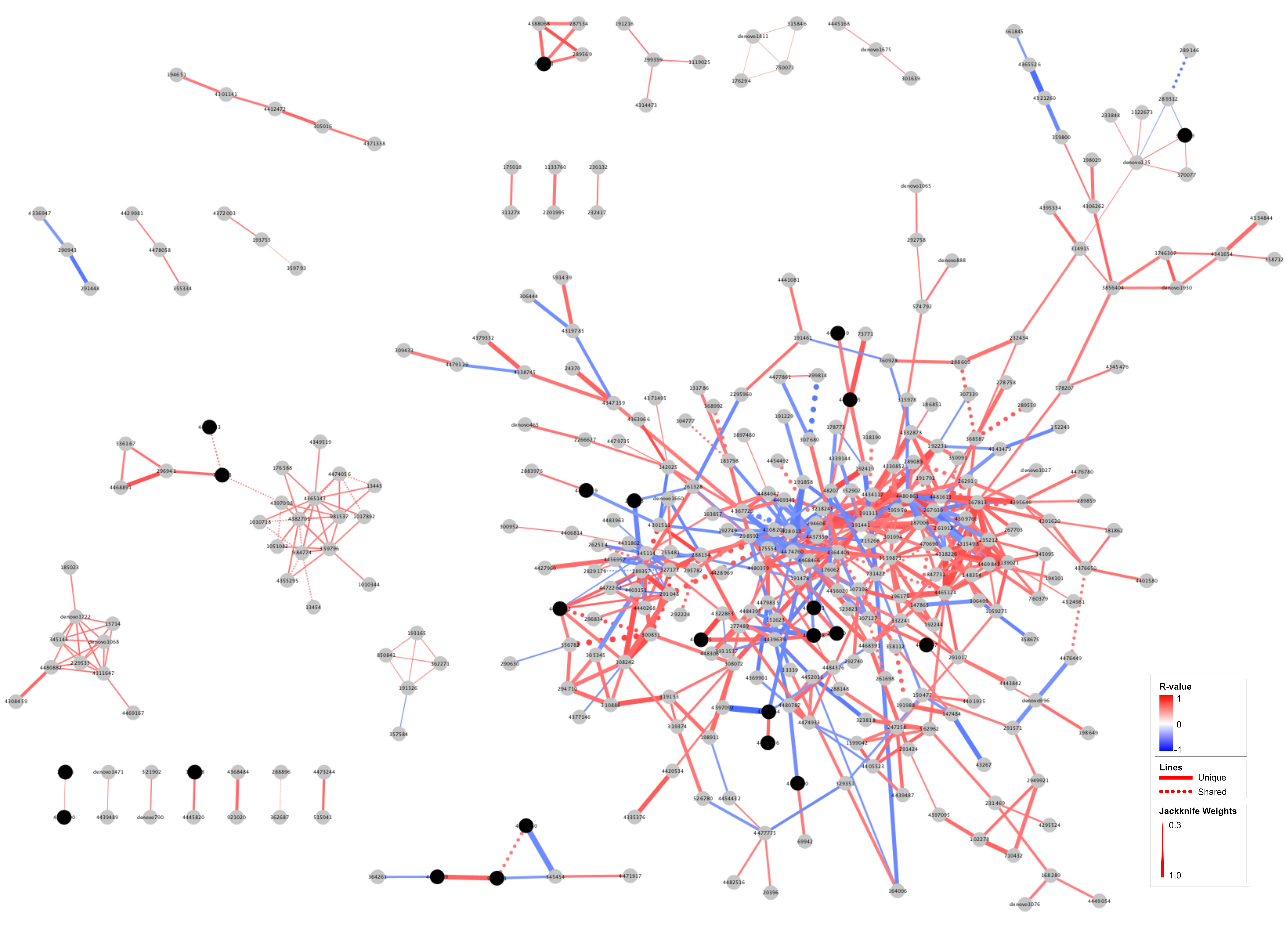

Supplement: Supplementary file 10 — Figure S4. Co-occurrence network of HIV-negative, Prevotella-rich MSM. Correlation network of the 345 OTUs in the 25 HIV-negative, Prevotella-rich MSM individuals calculated using SparCC. The value of the correlation is colored on the network edges with negative R-values in blue and positive in red. Only correlations greater than |0.5| are included in the network. Analysis was jackknifed 100 times on random subsets of 9 individuals. The edge weights from the jackknife are represented by line weight, with the thickest lines corresponding to correlations identified in all jackknife subsets. The network edges on this graph are compared to a network calculated using the same methods with a subset of 9 individuals that are HIV-negative, Prevotella-rich non-MSM. The edges that are shared between the MSM and non-MSM networks are dotted and the edges unique to the MSM network are solid. The OTUs identified in the Prevotella genus are colored black. (PNG 3690 kb) [file 40168_2018_580_MOESM10_ESM.png]

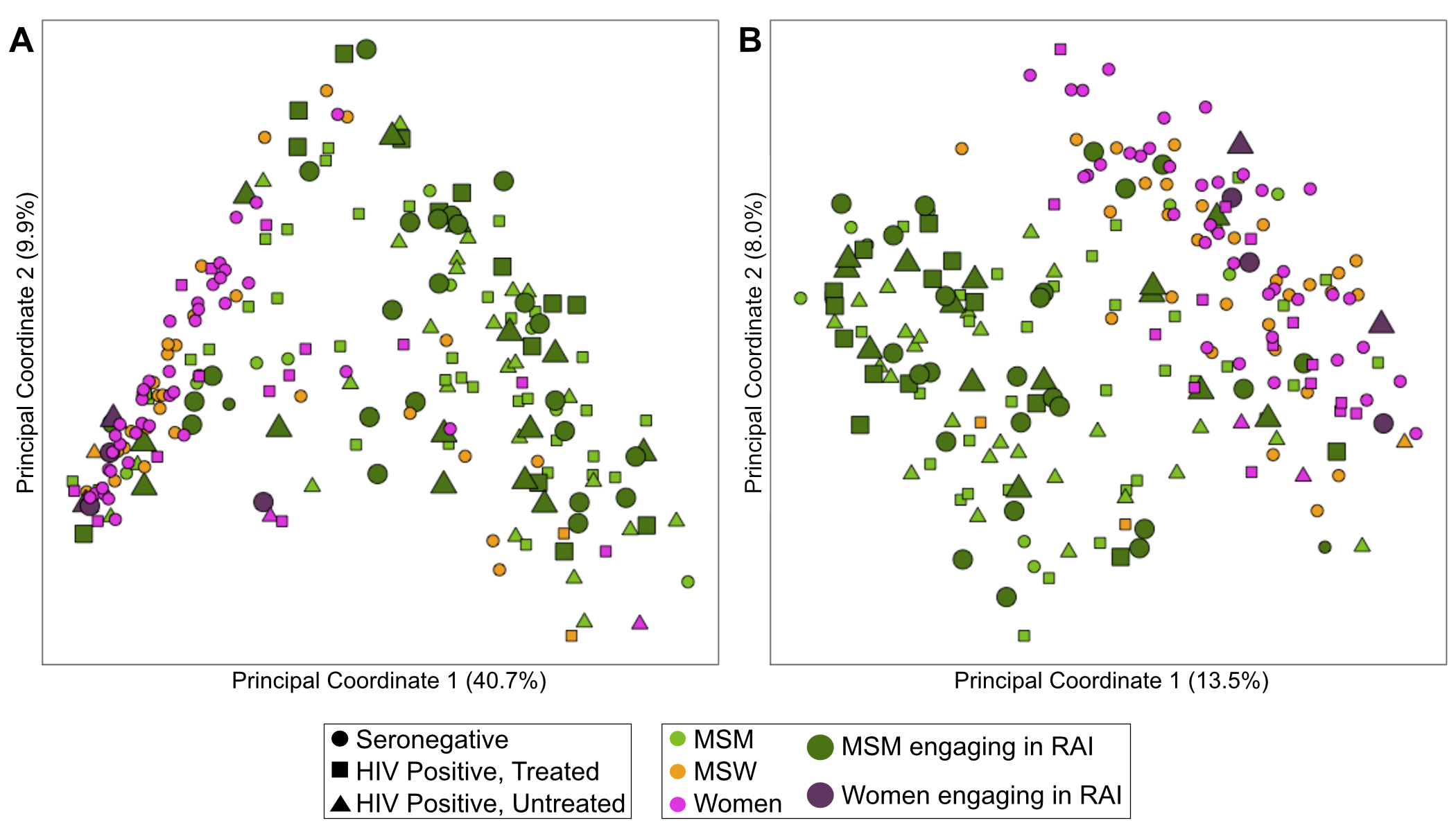

Supplement: Supplementary file 13 — Figure S5. Receptive anal intercourse does not correlate with PCoA space A. Weighted UniFrac PCoA and B. Unweighted UniFrac with points colored by orientation and shaped by HIV status. Women who reported engaging in RAI are colored dark purple and are larger in size than the other points. This plot highlights that women who engage in RAI cluster apart from the MSM with the other Bacteroides-rich women. (PNG 423 kb) [file 40168_2018_580_MOESM13_ESM.png]

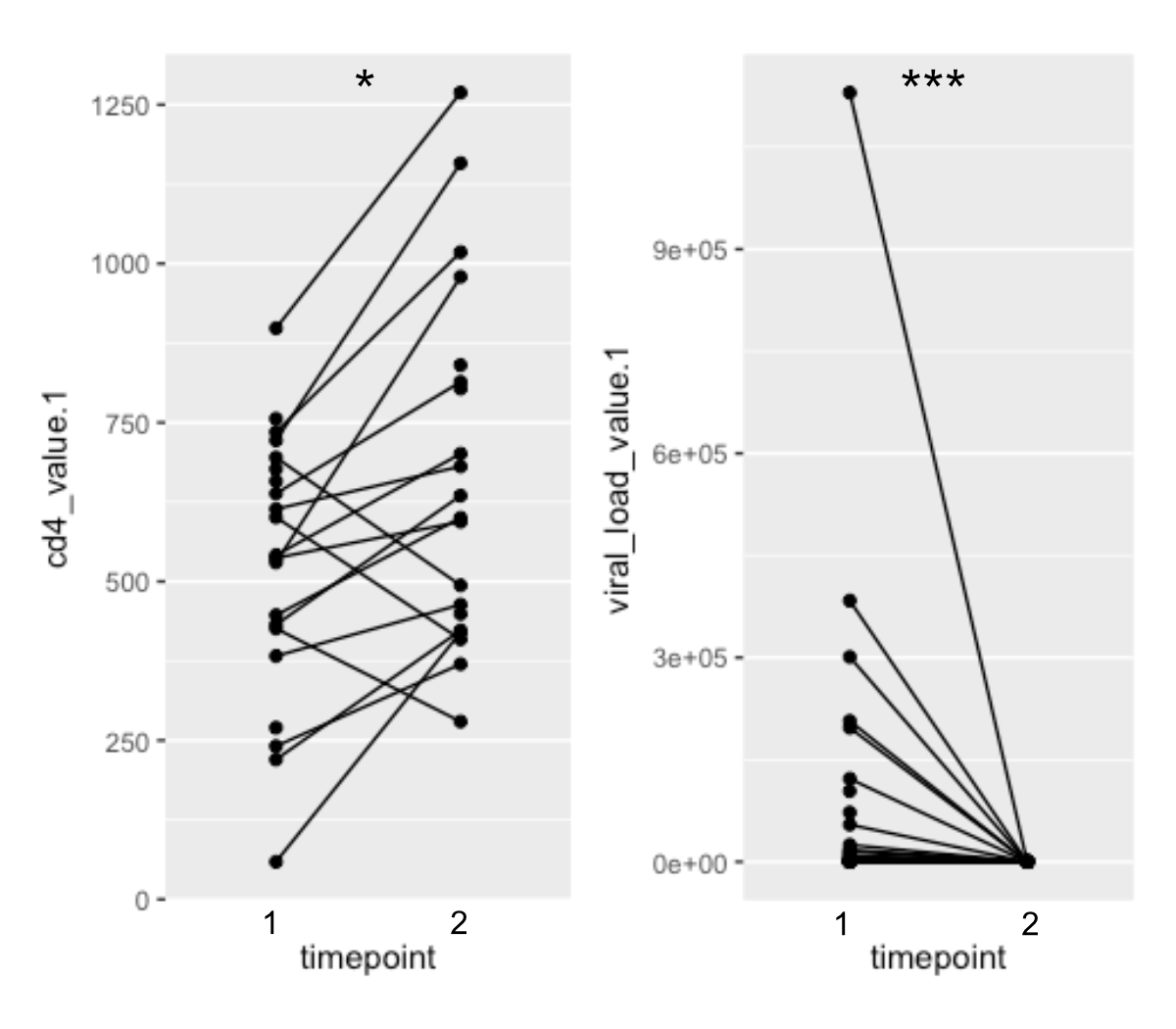

Supplement: Supplementary file 19 — Figure S6. HIV-positive individuals improve CD4+ T cell count and viral load after ART initiation. (Wilcoxon rank-sum; P-value: * < 0.05, *** < 0.001). (PNG 191 kb) [file 40168_2018_580_MOESM19_ESM.png]
