# Supplementary material for: An exploration of Prevotella-rich microbiomes in HIV and men who have sex with men
Source: Microbiome. 2018 Nov 5;6:198. doi: 10.1186/s40168-018-0580-7 (PMC6219090; doi:10.1186/s40168-018-0580-7)
Supplement: Supplementary file 20 — Markdown of analyses performed in this study. (PDF 274 kb) [file 40168_2018_580_MOESM20_ESM.pdf]

An exploration of Prevotella-rich microbiomes in HIV and men who have sex with men

Abigail JS Armstrong

6/12/2018

All QIIME analysis was performed using QIIME1.9.1

Sequencing Processing Overview

Quality filtering

6 MiSeq runs were demultiplexed separately in QIIME without any quality filtering. The resulting fastq files were run through DADA2 in R using inhouse code found at: github.com/shafferm/dada2_qiime1 All run reads were trimmed at 112 basepairs durrig DADA2 processing

OTU Picking

99% OTUs were picked on the DADA2 processed data using a combination of QIIME pipeline and inhouse scripts

Closed reference OTUs (sortmerna)

```
pick_otus.py -i ~/Data/HIV_6runs/OTUs/dada2_output/HIV_6_runs.fasta -C -m sortmerna
-s .99 --threads 3 -r ~/Desktop/blast_dbs/gg_13_8_otus/rep_set/99_otus.fasta
-o ~/Data/HIV_6runs/OTUs/99_otus/sortmerna_picked_otus/

filter_fasta.py -f ~/Data/HIV_6runs/OTUs/dada2_output/HIV_6_runs.fasta
-s ~/Data/HIV_6runs/OTUs/99_otus/sortmerna_picked_otus/HIV_6_runs_failures.txt
-o ~/Data/HIV_6runs/OTUs/99_otus/sortmerna_picked_otus/failures.fasta

pick_rep_set.py -i ~/Data/HIV_6runs/OTUs/99_otus/sortmerna_picked_otus/HIV_6_runs_otus.txt
-o ~/Data/HIV_6runs/OTUs/99_otus/sortmerna_picked_otus/rep_set.fna
-f ~/Data/HIV_6runs/OTUs/dada2_output/HIV_6_runs.fasta
```

Open reference OTUs (UCLUST)

```
pick_otus.py -i ~/Data/HIV_6runs/OTUs/99_otus/sortmerna_picked_otus/failures.fasta
-o ~/Data/HIV_6runs/OTUs/99_otus/open_ref_uclust/ -m uclust

pick_rep_set.py -i ~/Data/HIV_6runs/OTUs/99_otus/open_ref_uclust/failures_otus.txt
-o ~/Data/HIV_6runs/OTUs/99_otus/open_ref_uclust/failure_rep_set.fna
-f ~/Data/HIV_6runs/OTUs/99_otus/sortmerna_picked_otus/failures.fasta
```

Concatenating open and closed reference OTUs

```
cat ~/Data/HIV_6runs/OTUs/99_otus/sortmerna_picked_otus/rep_set.fna
~/Data/HIV_6runs/OTUs/99_otus/open_ref_uclust/failure_rep_set.fna >
~/Data/HIV_6runs/OTUs/99_otus/rep_set.fna

cat ~/Data/HIV_6runs/OTUs/99_otus/open_ref_uclust/failure_rep_set.fna >
```

```
~/Data/HIV_6runs/OTUs/99_otus/new_refseqs.fna

cat ~/Data/HIV_6runs/OTUs/99_otus/sortmerna_picked_otus/HIV_6_runs_otus.txt
~/Data/HIV_6runs/OTUs/99_otus/open_ref_uclust/failures_otus.txt >
~/Data/HIV_6runs/OTUs/99_otus/final_otu_map.txt
```

Creating an OTU table *code written my M. Shaffer*

```
from biom import load_table
from biom.table import Table
import numpy as np

otu_map = open("~/Data/HIV_6runs/OTUs/99_otus/final_otu_map.txt", 'U')
otu_map = otu_map.readlines()
otu_map = [i.strip().split() for i in otu_map]
otu_map = {i[0]: i[1:] for i in otu_map}

table = load_table("~/Data/HIV_6runs/OTUs/dada2_output/HIV_6_runs.biom")
new_table = np.zeros((len(otu_map), table.shape[1]))
for i, otu in enumerate(otu_map):
    for seq in otu_map[otu]:
        new_table[i,]+=table.data(seq, axis="observation")
new_table = Table(new_table, otu_map.keys(), table.ids())
new_table.to_json("dada2_to_otu_table",
    open("~/Data/HIV_6runs/OTUs/99_otus/otu_table.biom", 'w'))
```

Adding taxonomy

```
assign_taxonomy.py -o ~/Data/HIV_6runs/OTUs/99_otus/taxonomy/
-i ~/Data/HIV_6runs/OTUs/99_otus/rep_set.fna
-r ~/Desktop/blast_dbs/gg_13_8_otus/rep_set/99_otus.fasta
-t ~/Desktop/blast_dbs/gg_13_8_otus/taxonomy/99_otu_taxonomy.txt

biom add-metadata -i ~/Data/HIV_6runs/OTUs/99_otus/otu_table.biom
--observation-metadata-fp
~/Data/HIV_6runs/OTUs/99_otus/taxonomy/rep_set_tax_assignments.txt
-o ~/Data/HIV_6runs/OTUs/99_otus/otu_table_w_tax.biom --sc-separated taxonomy
--observation-header OTUID,taxonomy
```

Pynast alignment

```
align_seqs.py -i ~/Data/HIV_6runs/OTUs/99_otus/rep_set.fna
-o ~/Data/HIV_6runs/OTUs/99_otus/pynast_aligned_seqs/

filter_alignment.py -o ~/Data/HIV_6runs/OTUs/99_otus/pynast_aligned_seqs/
-i ~/Data/HIV_6runs/OTUs/99_otus/pynast_aligned_seqs/rep_set_aligned.fasta

make_phylogeny.py -i
~/Data/HIV_6runs/OTUs/99_otus/pynast_aligned_seqs/rep_set_aligned_pfiltered.fasta
-o ~/Data/HIV_6runs/OTUs/99_otus/rep_set.tree
```

Filtering pynast failures Code written by M. Shaffer

```
p = open("~/Data/HIV_6runs/OTUs/99_otus/pynast_aligned_seqs/failures.fasta")
f = p.readlines()
headers = [f[i].strip() for i in xrange(len(f)) if i%2==0]
```

```
ids_to_toss = [i[1:] for i in headers]

table = load_table("~/Data/HIV_6runs/OTUs/99_otus/otu_table.biom")
set_to_toss = set(table.ids(axis="observation")) & set(ids_to_toss)

table.filter(set_to_toss, invert=True, axis="observation")
table.to_json("remove_pynast_failures.py",
    open("~/Data/HIV_6runs/OTUs/99_otus/otu_table_no_pynase_failures.biom", 'w'))
```

Cohort Description

There were significantly higher CD4+ T cell numbers (cells/uL) in HIV-positive MSM on ART compared to the ART-naïve cohorts but no significant difference between ART-treated and ART-naïve HIV-positive women

```
library("ggplot2")
md <- read.table("~/Data/HIV_6runs/metadata/_R_metadata_one_rep_8-23.txt",
    header = TRUE, sep = "\t", strip.white = TRUE)
md.HIV <- subset(md, HIV == "Positive")
md.HIV.women <- subset(md.HIV, gender == "Female")
md.HIV.men <- subset(md.HIV, gender == "Male")
md.men <- subset(md, gender == "Male")
md.neg <- subset(md, HIV == "Negative")
```

CD4 and viral load stats HIV positive treated compared to untreated cohorts

```
kruskal.test(treatment ~ cd4_value, data = md.HIV)
```

```
##
##  Kruskal-Wallis rank sum test
##
## data:  treatment by cd4_value
## Kruskal-Wallis chi-squared = 106.88, df = 106, p-value = 0.4579
```

```
kruskal.test(treatment ~ viral_load_value, data = md.HIV)
```

```
##
##  Kruskal-Wallis rank sum test
##
## data:  treatment by viral_load_value
## Kruskal-Wallis chi-squared = 111, df = 57, p-value = 2.454e-05
```

HIV positive WOMEN treated compared to untreated cohorts

```
kruskal.test(treatment ~ cd4_value, data = md.HIV.women)
```

```
##
##  Kruskal-Wallis rank sum test
##
## data:  treatment by cd4_value
## Kruskal-Wallis chi-squared = 18, df = 18, p-value = 0.4557
```

```
kruskal.test(treatment ~ viral_load_value, data = md.HIV.women)
```

```
##
##  Kruskal-Wallis rank sum test
##
```

```
## data:  treatment by viral_load_value
## Kruskal-Wallis chi-squared = 18, df = 8, p-value = 0.02123
```

HIV positive MEN treated compared to untreated cohorts

```
kruskal.test(treatment ~ cd4_value, data = md.HIV.men)
```

```
##
##  Kruskal-Wallis rank sum test
##
## data:  treatment by cd4_value
## Kruskal-Wallis chi-squared = 92, df = 90, p-value = 0.4217
```

```
kruskal.test(treatment ~ viral_load_value, data = md.HIV.men)
```

```
##
##  Kruskal-Wallis rank sum test
##
## data:  treatment by viral_load_value
## Kruskal-Wallis chi-squared = 92, df = 51, p-value = 0.0003816
```

HIV Positive treated and untreaded subjects comparing men and women

```
kruskal.test(gender ~ viral_load_value, data = md.HIV)
```

```
##
##  Kruskal-Wallis rank sum test
##
## data:  gender by viral_load_value
## Kruskal-Wallis chi-squared = 46.357, df = 57, p-value = 0.8421
```

```
kruskal.test(gender ~ cd4_value, data = md.HIV)
```

```
##
##  Kruskal-Wallis rank sum test
##
## data:  gender by cd4_value
## Kruskal-Wallis chi-squared = 100.45, df = 106, p-value = 0.6339
```

CD4 count and Viral Load oh HIV positive subjects broken up by gender and treatment

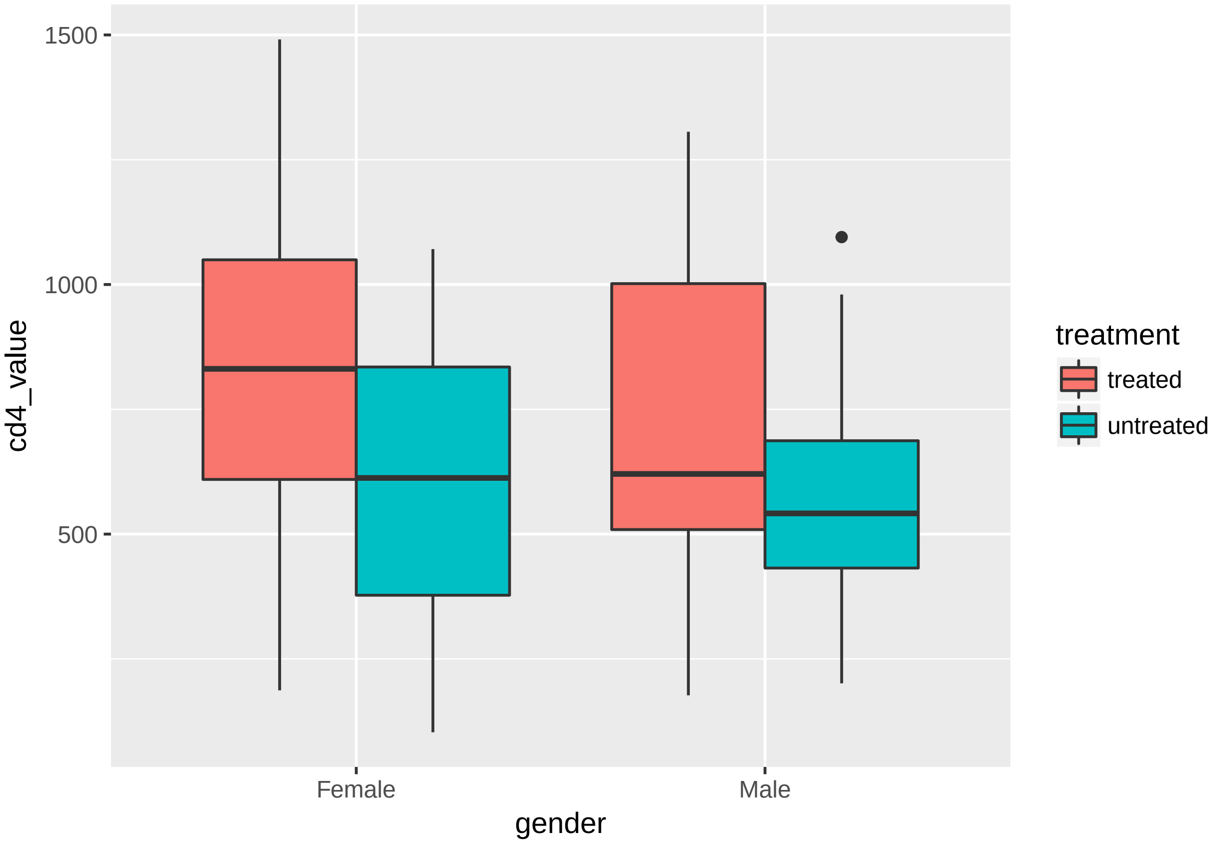
1500
1000
500
cd4_value
Female
Male
gender
treatment
treated
untreated

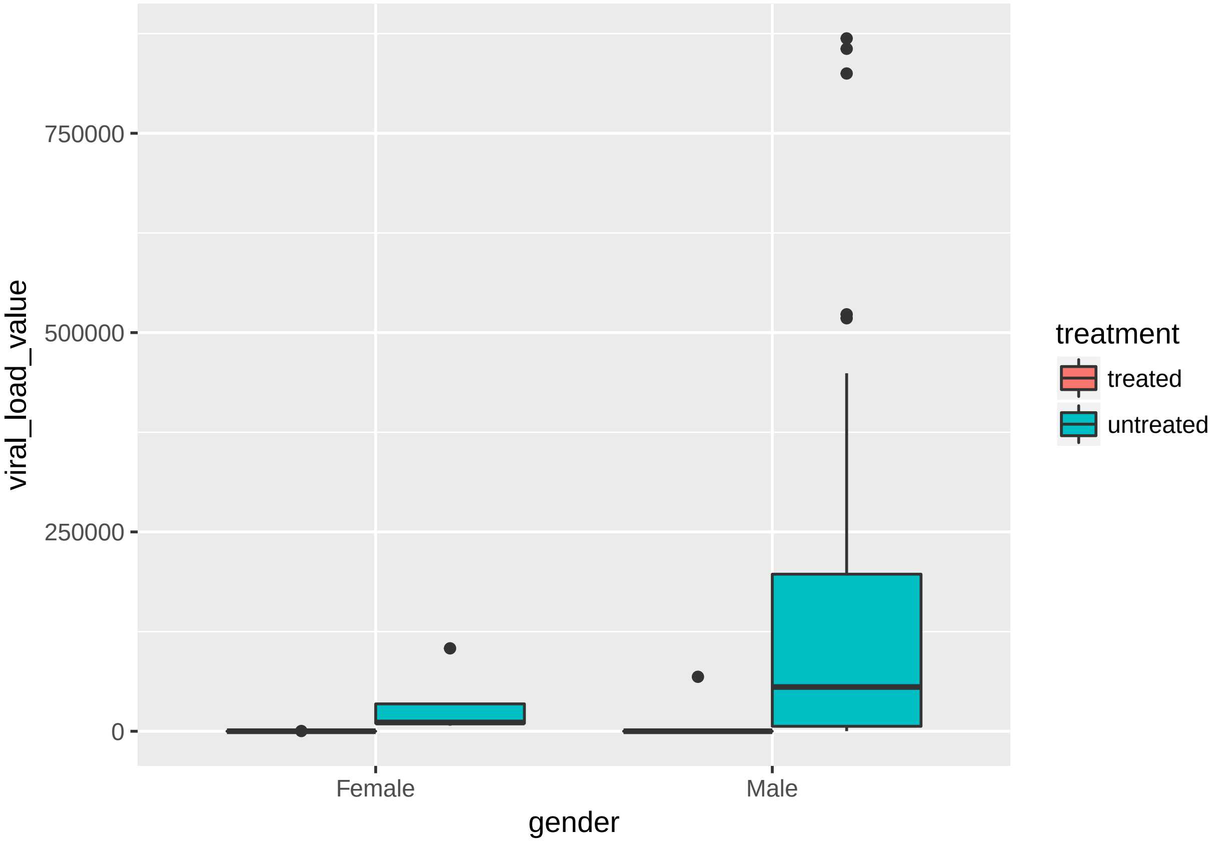
750000
500000
250000
0
viral_load_value
Female
Male
gender
treatment
treated
untreated

Sexual behavior is the strongest descriptor of compositional variation in the gut microbiome

Enterotyping

MSM were more likely to be in a Prevotella enterotype, as defined using partition around medoids (PAM) clustering

Code modified from http://enterotype.embl.de/enterotypes.html

```
data = read.table("~/Data/HIV_6runs/core_div/one_rep/table_even11218_L6_enterotype.txt",
    header = T, row.names = 1, dec = ".", sep = "\t")
data = data[-1, ]
```

Calculating Distance

```
JSD <- function(x, y) sqrt(0.5 * KLD(x, (x + y)/2) + 0.5 * KLD(y,
    (x + y)/2))
KLD <- function(x, y) sum(x * log(x/y))

dist.JSD <- function(inMatrix, pseudocount = 1e-06, ...) {
    KLD <- function(x, y) sum(x * log(x/y))
    JSD <- function(x, y) sqrt(0.5 * KLD(x, (x + y)/2) + 0.5 *
        KLD(y, (x + y)/2))
    matrixColSize <- length(colnames(inMatrix))
    matrixRowSize <- length(rownames(inMatrix))
    colnames <- colnames(inMatrix)
    resultsMatrix <- matrix(0, matrixColSize, matrixColSize)

    inMatrix = apply(inMatrix, 1:2, function(x) ifelse(x == 0,
        pseudocount, x))

    for (i in 1:matrixColSize) {
        for (j in 1:matrixColSize) {
            resultsMatrix[i, j] = JSD(as.vector(inMatrix[, i]),
                as.vector(inMatrix[, j]))
        }
    }
    rownames(resultsMatrix) <- colnames(resultsMatrix) <- colnames
    resultsMatrix <- as.dist(resultsMatrix)
    attr(resultsMatrix, "method") <- "dist"
    return(resultsMatrix)
}

data.dist = dist.JSD(data)
```

Defining clusters

```
k = 2
library(cluster)

# x is a distance matrix and k the number of clusters
pam.clustering = function(x, k) {
    require(cluster)
    cluster = as.vector(pam(as.dist(x), k, diss = TRUE)$clustering)
    return(cluster)
```

```
}

data.cluster = pam.clustering(data.dist, k = k)
```

Determining optimal number of clusters

```
require(clusterSim)
```

```
## Loading required package: clusterSim
```

```
## Loading required package: MASS
```

```
##
## This is package 'modeest' written by P. PONCET.
## For a complete list of functions, use 'library(help = "modeest")' or 'help.start()'.
```

```
nclusters = index.G1(t(data), data.cluster, d = data.dist, centrotypes = "medoids")

nclusters = NULL

for (k.iter in 1:20) {
    if (k.iter == 1) {
        nclusters[k.iter] = NA
    } else {
        data.cluster.temp = pam.clustering(data.dist, k.iter)
        nclusters[k.iter] = index.G1(t(data), data.cluster.temp,
            d = data.dist, centrotypes = "medoids")
    }
}

plot(nclusters, type = "h", xlab = "k clusters", ylab = "CH index")
```

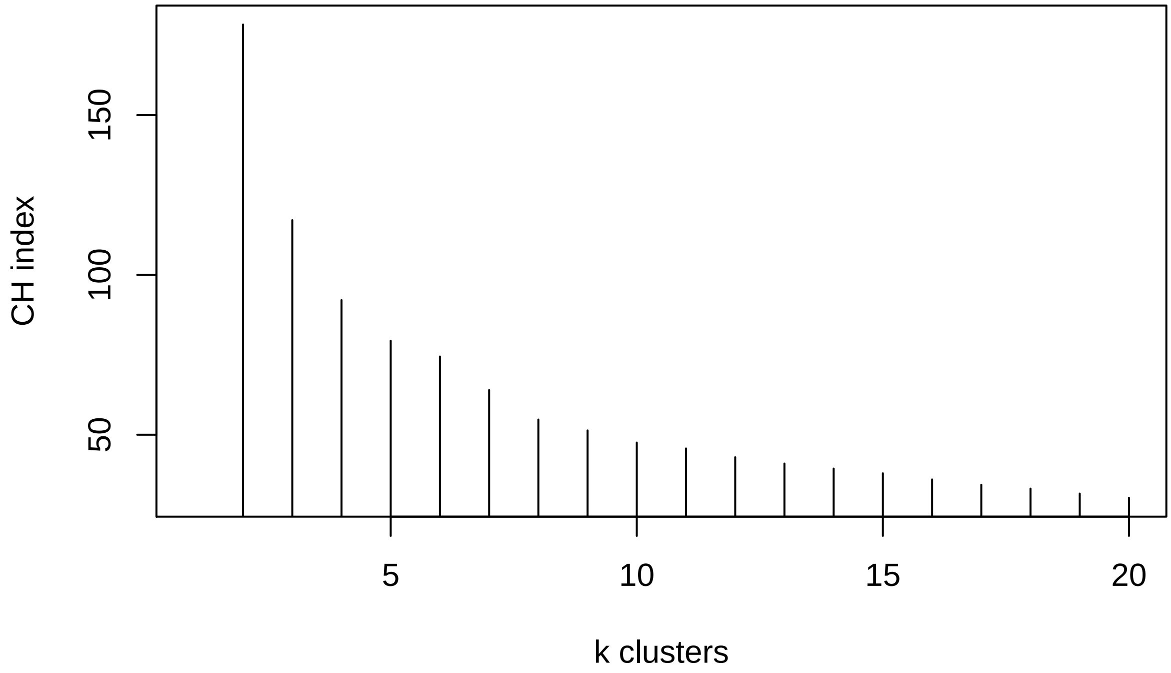


```
obs.silhouette = mean(silhouette(data.cluster, data.dist)[, 3])
```

Noise Removal

```
noise.removal <- function(dataframe, percent = 0.01, top = NULL) {
    Matrix <- dataframe
    bigones <- rowSums(Matrix) * 100/(sum(rowSums(Matrix))) >
        percent
    Matrix_1 <- Matrix[bigones, ]
    print(percent)
    return(Matrix_1)
}
data.denoized = noise.removal(data, percent = 0.01)
```

```
## [1] 0.01
```

Between Class Analysis

```
library(ade4)
obs.pca = dudi.pca(data.frame(t(data)), scannf = F, nf = 10)
obs.bet = bca(obs.pca, fac = as.factor(data.cluster), scannf = F,
    nf = k - 1)
s.class(obs.bet$ls, fac = as.factor(data.cluster), grid = F)
s.class(obs.bet$ls, fac = as.factor(data.cluster), grid = F,
    cell = 0, cstar = 0, col = c(4, 2, 3))
```

PCoA Analaysis

```
obs.pcoa = dudi.pco(data.dist, scannf = F, nf = 3)
s.class(obs.pcoa$li, fac = as.factor(data.cluster), grid = F)
s.class(obs.pcoa$li, fac = as.factor(data.cluster), grid = F,
    cell = 0, cstar = 0, col = c(3, 2, 4))
```

Enterotyping statistics

Fishers test independence of orientation and enterotype

```
library(rcompanion)
enterotype.tbl <- table(md$MSM, md$Enterotype)
pairwiseNominalIndependence(as.matrix(enterotype.tbl), fisher = TRUE,
    gtest = FALSE, chisq = FALSE, digits = 3)
```

```
##      Comparison p.Fisher p.adj.Fisher
## 1 Female : MSM 3.86e-19     1.16e-18
## 2 Female : MSW 3.72e-02     3.72e-02
## 3    MSM : MSW 3.05e-07     4.57e-07
```

Adonis test of the weighted and unweighted unifrac

Applying the Adonis test showed significant effects for MSM and HIV with weighted Unifrac, which explained 16.4% and 1.4% of the variation respectively, and for MSM, HIV, and ART with unweighted Unifrac, which explained 7.0%, 0.9%, 0.7% respectively files/packages

```
library(vegan)
```

```
## Loading required package: permute

## Loading required package: lattice

## This is vegan 2.4-4
```

```r
dist.file.wu <- "~/Data/HIV_6runs/core_div/one_rep/bdiv_even11218/weighted_unifrac_dm.txt"
dist.matrix.wu <- as.matrix(read.table(dist.file.wu, sep = "\t",
    header = T, row.names = 1, check = FALSE, quote = "\""))

dist.file.uwu <- "~/Data/HIV_6runs/core_div/one_rep/bdiv_even11218/unweighted_unifrac_dm.txt"
dist.matrix.uwu <- as.matrix(read.table(dist.file.uwu, sep = "\t",
    header = T, row.names = 1, check = FALSE, quote = "\""))
```

Running adonis test - running 10000 permutations resulted in the most consistent results. However results may vary slightly from published runs of permuted adonis.

```r
adonis(as.dist(dist.matrix.wu) ~ MSM + HIV + treatment, data = md,
    permutations = 10000)
```

```
##
## Call:
## adonis(formula = as.dist(dist.matrix.wu) ~ MSM + HIV + treatment,      data = md, permutations = 100[illegible]
##
## Permutation: free
## Number of permutations: 10000
##
## Terms added sequentially (first to last)
##
##            Df SumsOfSqs MeanSqs F.Model      R2    Pr(>F)
## MSM         2    2.4578 1.22890 21.2134 0.16374 9.999e-05 ***
## HIV         1    0.2042 0.20421  3.5251 0.01360    0.0105 *
## treatment   1    0.0672 0.06720  1.1600 0.00448    0.2705
## Residuals 212   12.2813 0.05793         0.81818
## Total     216   15.0105                 1.00000
## ---
## Signif. codes:  0 '***' 0.001 '**' 0.01 '*' 0.05 '.' 0.1 ' ' 1
```

```r
adonis(as.dist(dist.matrix.uwu) ~ MSM + HIV + treatment, data = md,
    permutations = 10000)
```

```
##
## Call:
## adonis(formula = as.dist(dist.matrix.uwu) ~ MSM + HIV + treatment,      data = md, permutations = 10[illegible]
##
## Permutation: free
## Number of permutations: 10000
##
## Terms added sequentially (first to last)
##
##            Df SumsOfSqs MeanSqs F.Model      R2    Pr(>F)
## MSM         2     2.746 1.37291  8.1973 0.07064 9.999e-05 ***
## HIV         1     0.338 0.33814  2.0189 0.00870  0.005399 **
## treatment   1     0.278 0.27842  1.6624 0.00716  0.021398 *
## Residuals 212    35.507 0.16748         0.91349
## Total     216    38.869                 1.00000
## ---
## Signif. codes:  0 '***' 0.001 '**' 0.01 '*' 0.05 '.' 0.1 ' ' 1
```

Comparison of MSM and non-MSM

HIV-negative and HIV-positive MSM had significantly greater Phylogenetic Diversity (PD; (30)) and observed Operational Taxonomic Units (OTUs, defined at a 99%) compared to non-MSM and many significantly different OTUs and genera.

Alpha diversity statistics

Data

```
alpa.div <- read.table("~/Data/HIV_6runs/stats/alpha_div/alpha_div.txt",
    header = TRUE, sep = "\t", strip.white = TRUE)
alpha.div.HIV.pos <- subset(alpa.div, HIV == "Positive")
alpa.div.HIV.neg <- subset(alpa.div, HIV == "Negative")
```

Stats on HIV Positive Subjects

```
kruskal.test(PD_whole_tree ~ MSM, data = alpha.div.HIV.pos)
```

```
##
##  Kruskal-Wallis rank sum test
##
## data:  PD_whole_tree by MSM
## Kruskal-Wallis chi-squared = 9.64, df = 2, p-value = 0.008067
```

```
kruskal.test(observed_otus ~ MSM, data = alpha.div.HIV.pos)
```

```
##
##  Kruskal-Wallis rank sum test
##
## data:  observed_otus by MSM
## Kruskal-Wallis chi-squared = 9.5712, df = 2, p-value = 0.008349
```

```
kruskal.test(shannon ~ MSM, data = alpha.div.HIV.pos)
```

```
##
##  Kruskal-Wallis rank sum test
##
## data:  shannon by MSM
## Kruskal-Wallis chi-squared = 10.423, df = 2, p-value = 0.005454
```

Stats on HIV Negative Subjects

```
kruskal.test(PD_whole_tree ~ MSM, data = alpa.div.HIV.neg)
```

```
##
##  Kruskal-Wallis rank sum test
##
## data:  PD_whole_tree by MSM
## Kruskal-Wallis chi-squared = 3.4326, df = 2, p-value = 0.1797
```

```
kruskal.test(observed_otus ~ MSM, data = alpa.div.HIV.neg)
```

```
##
##  Kruskal-Wallis rank sum test
##
## data:  observed_otus by MSM
## Kruskal-Wallis chi-squared = 3.4775, df = 2, p-value = 0.1757
```

```r
kruskal.test(shannon ~ MSM, data = alpa.div.HIV.neg)
```

```
##
##  Kruskal-Wallis rank sum test
##
## data:  shannon by MSM
## Kruskal-Wallis chi-squared = 0.43477, df = 2, p-value = 0.8046
```

OTU difference statustics were calculated in QIIME 1.9.1

OTU table was filtered for only OTUs present in at lease 20% of the samples

```
filter_otus_from_otu_table.py
-i ~/Data/HIV_6runs/stats/MSM_comp/otu_table_neg_RA.biom
-o ~/Data/HIV_6runs/stats/MSM_comp/otu_table_neg_RA_filt20.biom
-s 13

group_significance.py
-i ~/Data/HIV_6runs/stats/MSM_comp/otu_table_neg_RA_filt20.biom
-o ~/Data/HIV_6runs/stats/MSM_comp/kw_otus.txt
-m ~/Data/HIV_6runs/metadata/_metadata_one_rep_6_30.txt
-c MSMyn
```

Prevotella-rich microbiomes differ in MSM compared to non-MSM

We found that the proportion of Prevotella-rich individuals who did not fit within the Prevotella reference space was significantly higher for MSM (81.4% of individuals) than non-MSM (46.7%) Enterotyping fit was performed using the tools outlined at enterotypes.org

```r
md.prev <- subset(md, Enterotype == "Prevotella")
prev.ent.fit.tbl <- table(md.prev$MSMyn, md.prev$Within_ET_space)
pairwiseNominalIndependence(as.matrix(prev.ent.fit.tbl), fisher = TRUE,
    gtest = FALSE, chisq = FALSE, digits = 3)
```

```
##   Comparison p.Fisher p.adj.Fisher
## 1   No : Yes  0.00641      0.00641
```

Prevotella-rich, HIV-negative MSM (n=25) and non-MSM (n=9) had significantly different relative abundances of 39 OTUs in 21 different genera OTU difference statustics were calculated in QIIME 1.9.1

OTU table was filtered for only OTUs present in at lease 20% of the samples

```
filter_otus_from_otu_table.py
-i ~/Data/HIV_6runs/stats/HIV_neg/otu_table_neg_RA.biom
-o ~/Data/HIV_6runs/stats/HIV_neg/otu_table_neg_RA_filt20.biom
-s 13

group_significance.py
-i ~/Data/HIV_6runs/stats/HIV_neg/otu_table_neg_RA_filt20.biom
-o ~/Data/HIV_6runs/stats/HIV_neg/kw_otus.txt
-m ~/Data/HIV_6runs/metadata/_metadata_one_rep_6_30.txt
-c MSMyn
```

Potential drivers of a Prevotella-enterotype in MSM

Receptive Anal intercourse

Taxonomy test peformed in QIIME 1.9.1

We did not find any significant association with microbiome taxonomy or alpha or beta diversity between MSM who engaged in RAI ($n = 31$) and those who did not ($n = 16$) when controlling for sexual orientation and HIV status (taxonomy and alpha diversity – Kruskal-Wallis test; beta diversity – adonis test). HIV negative MSM

```
group_significance.py
-i ~/Data/HIV_6runs/stats/HIV_neg_MSM/otu_table_neg_RA_filt20.biom
-o ~/Data/HIV_6runs/stats/HIV_neg_MSM/kw_otus.txt
-m ~/Data/HIV_6runs/metadata/_metadata_one_rep_6_30.txt
-c RAI
```

HIV positive MSM

```
group_significance.py
-i ~/Data/HIV_6runs/stats/HIV_pos_MSM/otu_table_neg_RA_filt20.biom
-o ~/Data/HIV_6runs/stats/HIV_pos_MSM/kw_otus.txt
-m ~/Data/HIV_6runs/metadata/_metadata_one_rep_6_30.txt
-c RAI
```

All MSM

```
group_significance.py
-i ~/Data/HIV_6runs/stats/all_MSM/otu_table_neg_RA_filt20.biom
-o ~/Data/HIV_6runs/stats/all_MSM/kw_otus.txt
-m ~/Data/HIV_6runs/metadata/_metadata_one_rep_6_30.txt
-c RAI
```

Alpha diversity

Files

```
inf.MSM <- "~/Data/HIV_6runs/stats/MSM_only/alpha_div.txt"
df.MSM <- read.table(inf.MSM, header = TRUE, sep = "\t", strip.white = TRUE)

df.MSM.pos <- subset(df.MSM, HIV == "Positive")
df.MSM.neg <- subset(df.MSM, HIV == "Negative")
```

HIV negative MSM

```
kruskal.test(observed_otus ~ RAI_frequency, data = df.MSM.neg)
```

```
##
##  Kruskal-Wallis rank sum test
##
## data:  observed_otus by RAI_frequency
## Kruskal-Wallis chi-squared = 0.079684, df = 2, p-value = 0.9609
```

```
kruskal.test(shannon ~ RAI_frequency, data = df.MSM.neg)
```

```
##
##  Kruskal-Wallis rank sum test
##
## data:  shannon by RAI_frequency
```

```
## Kruskal-Wallis chi-squared = 0.26563, df = 2, p-value = 0.8756
```

HIV positve MSM

```
kruskal.test(observed_otus ~ RAI_frequency, data = df.MSM.pos)
```

```
##
##  Kruskal-Wallis rank sum test
##
## data:  observed_otus by RAI_frequency
## Kruskal-Wallis chi-squared = 0.48558, df = 2, p-value = 0.7844
```

```
kruskal.test(shannon ~ RAI_frequency, data = df.MSM.pos)
```

```
##
##  Kruskal-Wallis rank sum test
##
## data:  shannon by RAI_frequency
## Kruskal-Wallis chi-squared = 0.78811, df = 2, p-value = 0.6743
```

All MSM

```
kruskal.test(observed_otus ~ RAI_frequency, data = df.MSM)
```

```
##
##  Kruskal-Wallis rank sum test
##
## data:  observed_otus by RAI_frequency
## Kruskal-Wallis chi-squared = 0.090442, df = 2, p-value = 0.9558
```

```
kruskal.test(shannon ~ RAI_frequency, data = df.MSM)
```

```
##
##  Kruskal-Wallis rank sum test
##
## data:  shannon by RAI_frequency
## Kruskal-Wallis chi-squared = 0.19657, df = 2, p-value = 0.9064
```

Beta diversity

HIV negative MSM

Weighted

```
dist.file.wu.MSM.neg <- "~/Data/HIV_6runs/stats/MSM_only/beta_div/weighted_unifrac_MSM_negative.txt"
dist.matrix.wu.MSM.neg <- as.matrix(read.table(dist.file.wu.MSM.neg,
    sep = "\t", header = T, row.names = 1, check = FALSE, quote = "\""))

adonis(as.dist(dist.matrix.wu.MSM.neg) ~ RAI_frequency, data = df.MSM.neg,
    permutations = 10000)
```

Unweighted

```
dist.file.uwu.MSM.neg <- "~/Data/HIV_6runs/stats/MSM_only/beta_div/unweighted_unifrac_MSM_negative.txt"
dist.matrix.uwu.MSM.neg <- as.matrix(read.table(dist.file.uwu.MSM.neg,
    sep = "\t", header = T, row.names = 1, check = FALSE, quote = "\""))

adonis(as.dist(dist.matrix.uwu.MSM.neg) ~ RAI_frequency, data = df.MSM.neg,
    permutations = 10000)
```

HIV positve MSM

Weighted

```
dist.file.wu.MSM.pos <- "~/Data/HIV_6runs/stats/MSM_only/beta_div/weighted_unifrac_MSM_positive.txt"
dist.matrix.wu.MSM.pos <- as.matrix(read.table(dist.file.wu.MSM.pos,
    sep = "\t", header = T, row.names = 1, check = FALSE, quote = "\""))

adonis(as.dist(dist.matrix.wu.MSM.pos) ~ RAI_frequency, data = df.MSM.pos,
    permutations = 10000)
```

Unweighted

```
dist.file.uwu.MSM.pos <- "~/Data/HIV_6runs/stats/MSM_only/beta_div/unweighted_unifrac_MSM_positive.txt"
dist.matrix.uwu.MSM.pos <- as.matrix(read.table(dist.file.uwu.MSM.pos,
    sep = "\t", header = T, row.names = 1, check = FALSE, quote = "\""))

adonis(as.dist(dist.matrix.uwu.MSM.pos) ~ RAI_frequency, data = df.MSM.pos,
    permutations = 10000)
```

All MSM

Weighted

```
dist.file.wu.MSM <- "~/Data/HIV_6runs/stats/MSM_only/beta_div/weighted_unifrac_MSM.txt"
dist.matrix.wu.MSM <- as.matrix(read.table(dist.file.wu.MSM,
    sep = "\t", header = T, row.names = 1, check = FALSE, quote = "\""))

adonis(as.dist(dist.matrix.wu.MSM) ~ RAI_frequency, data = df.MSM,
    permutations = 10000)
```

Unweighted

```
dist.file.uwu.MSM <- "~/Data/HIV_6runs/stats/MSM_only/beta_div/unweighted_unifrac_MSM.txt"
dist.matrix.uwu.MSM <- as.matrix(read.table(dist.file.uwu.MSM,
    sep = "\t", header = T, row.names = 1, check = FALSE, quote = "\""))

adonis(as.dist(dist.matrix.uwu.MSM) ~ RAI_frequency, data = df.MSM,
    permutations = 10000)
```

Diet

Comparison of diets between HIV-negative MSM (n = 24) and non-MSM (n = 45) revealed several significant differences in diet composition

```
group_significance.py
-i ~/Data/HIV_6runs/diet_data/diet_kcalnorm_MPED.txt
-o ~/Data/HIV_6runs/diet_data/diet_diferences.txt
-m ~/Data/HIV_6runs/metadata/_metadata_one_rep_6_30.txt
-c MSM
```

Identifying HIV-associated microbiome differences while controlling for sexual behavior and gender.

Beta Diversity

This clustering pattern was not explained by CD4+ T cell count, viral load, CD4 nadir, CD4+CD38+HLA-DR+ and CD8+CD38+HLA-DR+ cells, antibiotic use in past 6 months, and engagement in RAI.

Files

```
inf.women <- "~/Data/HIV_6runs/stats/women_only/nadir/all_women_metadata.txt"
df.women <- read.table(inf.women, header = TRUE, sep = "\t",
    strip.white = TRUE)

df.women.cd4 <- subset(df.women, cd4_value >= 0)
df.women.pos <- subset(df.women, HIV == "Positive")
```

CD4+ T cell count

```
cor.test(df.women$wu_PC1_women, df.women$cd4_value, method = "spearman")
```

```
##
##  Spearman's rank correlation rho
##
## data:  df.women$wu_PC1_women and df.women$cd4_value
## S = 988, p-value = 0.08
## alternative hypothesis: true rho is not equal to 0
## sample estimates:
##        rho
## -0.4529412
```

```
cor.test(df.women$wu_PC2_women, df.women$cd4_value, method = "spearman")
```

```
##
##  Spearman's rank correlation rho
##
## data:  df.women$wu_PC2_women and df.women$cd4_value
## S = 542, p-value = 0.4496
## alternative hypothesis: true rho is not equal to 0
## sample estimates:
##       rho
## 0.2029412
```

Viral Load

```
cor.test(df.women.pos$wu_PC1_women, df.women.pos$viral_load_value,
    method = "spearman")
```

```
## Warning in cor.test.default(df.women.pos$wu_PC1_women, df.women.pos
## $viral_load_value, : Cannot compute exact p-value with ties
```

```
##
##  Spearman's rank correlation rho
##
## data:  df.women.pos$wu_PC1_women and df.women.pos$viral_load_value
## S = 794.74, p-value = 0.5322
## alternative hypothesis: true rho is not equal to 0
## sample estimates:
##        rho
## -0.1687323
```

```
cor.test(df.women.pos$wu_PC2_women, df.women.pos$viral_load_value,
    method = "spearman")
```

```
## Warning in cor.test.default(df.women.pos$wu_PC2_women, df.women.pos
## $viral_load_value, : Cannot compute exact p-value with ties
```

```
##
##  Spearman's rank correlation rho
##
## data:  df.women.pos$wu_PC2_women and df.women.pos$viral_load_value
## S = 916.78, p-value = 0.1863
## alternative hypothesis: true rho is not equal to 0
## sample estimates:
##        rho
## -0.3482021
```

CD4 nadir

```
cor.test(df.women$wu_PC1_women, df.women$cd4_nadir, method = "spearman")
```

```
##
##  Spearman's rank correlation rho
##
## data:  df.women$wu_PC1_women and df.women$cd4_nadir
## S = 282, p-value = 0.459
## alternative hypothesis: true rho is not equal to 0
## sample estimates:
##       rho
## 0.2252747
```

```
cor.test(df.women$wu_PC2_women, df.women$cd4_nadir, method = "spearman")
```

```
##
##  Spearman's rank correlation rho
##
## data:  df.women$wu_PC2_women and df.women$cd4_nadir
## S = 416, p-value = 0.6428
## alternative hypothesis: true rho is not equal to 0
## sample estimates:
##        rho
## -0.1428571
```

CD4+CD38+HLA-DR+ cells

```
cor.test(df.women$wu_PC1_women, df.women$CD4.HLADR_CD38_percent,
    method = "spearman")
```

```
## Warning in cor.test.default(df.women$wu_PC1_women, df.women
## $CD4.HLADR_CD38_percent, : Cannot compute exact p-value with ties
```

```
##
##  Spearman's rank correlation rho
##
## data:  df.women$wu_PC1_women and df.women$CD4.HLADR_CD38_percent
## S = 3000.8, p-value = 0.6769
## alternative hypothesis: true rho is not equal to 0
## sample estimates:
##        rho
## 0.08401602
```

```r
cor.test(df.women$wu_PC2_women, df.women$CD4.HLADR_CD38_percent,
    method = "spearman")
```

```
## Warning in cor.test.default(df.women$wu_PC2_women, df.women
## $CD4.HLADR_CD38_percent, : Cannot compute exact p-value with ties
```

```
##
##  Spearman's rank correlation rho
##
## data:  df.women$wu_PC2_women and df.women$CD4.HLADR_CD38_percent
## S = 4399.5, p-value = 0.07989
## alternative hypothesis: true rho is not equal to 0
## sample estimates:
##        rho
## -0.3429609
```

CD8+CD38+HLA-DR+ cells

```r
cor.test(df.women$wu_PC1_women, df.women$CD8.HLADR_CD38_percent,
    method = "spearman")
```

```
## Warning in cor.test.default(df.women$wu_PC1_women, df.women
## $CD8.HLADR_CD38_percent, : Cannot compute exact p-value with ties
```

```
##
##  Spearman's rank correlation rho
##
## data:  df.women$wu_PC1_women and df.women$CD8.HLADR_CD38_percent
## S = 3459.8, p-value = 0.781
## alternative hypothesis: true rho is not equal to 0
## sample estimates:
##         rho
## -0.05611835
```

```r
cor.test(df.women$wu_PC2_women, df.women$CD8.HLADR_CD38_percent,
    method = "spearman")
```

```
## Warning in cor.test.default(df.women$wu_PC2_women, df.women
## $CD8.HLADR_CD38_percent, : Cannot compute exact p-value with ties
```

```
##
##  Spearman's rank correlation rho
##
## data:  df.women$wu_PC2_women and df.women$CD8.HLADR_CD38_percent
## S = 4127.9, p-value = 0.1902
## alternative hypothesis: true rho is not equal to 0
## sample estimates:
##        rho
## -0.2600457
```

antibiotic use in past 6 months

```r
df.women.abx <- subset(df.women, antibiotics_past_6_months !=
    "NA")
kruskal.test(wu_PC1_women ~ antibiotics_past_6_months, data = df.women.abx)
```

```
##
##  Kruskal-Wallis rank sum test
```

```
##
## data:  wu_PC1_women by antibiotics_past_6_months
## Kruskal-Wallis chi-squared = 5.6415, df = 1, p-value = 0.01754
```

```
kruskal.test(wu_PC2_women ~ antibiotics_past_6_months, data = df.women.abx)
```

```
##
##  Kruskal-Wallis rank sum test
##
## data:  wu_PC2_women by antibiotics_past_6_months
## Kruskal-Wallis chi-squared = 0.58435, df = 1, p-value = 0.4446
```

engagement in RAI

```
df.women.RAI <- subset(df.women, RAI_frequency != "NA")
kruskal.test(wu_PC1_women ~ RAI_frequency, data = df.women)
```

```
##
##  Kruskal-Wallis rank sum test
##
## data:  wu_PC1_women by RAI_frequency
## Kruskal-Wallis chi-squared = 3.6182, df = 2, p-value = 0.1638
```

```
kruskal.test(wu_PC2_women ~ RAI_frequency, data = df.women)
```

```
##
##  Kruskal-Wallis rank sum test
##
## data:  wu_PC2_women by RAI_frequency
## Kruskal-Wallis chi-squared = 5.6306, df = 2, p-value = 0.05989
```

Longitudinal analysis of HIV-positive individuals before and after ART initiation

Files

```
inf.longitudinal <- "~/Data/HIV_6runs/stats/longitudinal/metadata_long_R.txt"
df.long <- read.table(inf.longitudinal, header = TRUE, sep = "\t",
    strip.white = TRUE)
df.wide <- reshape(data = df.long, timevar = "timepoint", direction = "wide",
    idvar = c("HIV_ID", "HIV", "group", "MSM", "gender", "race",
        "ethnicity", "uwu_distance", "wu_distance"))
```

Delta calculations

Time

```
df.wide$timedif.days <- as.Date(df.wide$study_visit_date.2, format = "%m/%d/%y") -
    as.Date(df.wide$study_visit_date.1, format = "%m/%d/%y")
```

```
## Warning in strptime(x, format, tz = "GMT"): unknown timezone 'zone/tz/
## 2018c.1.0/zoneinfo/America/Denver'
```

```
df.wide$timedif.months <- as.numeric(df.wide$timedif.days)/30.4
```

Alpha diversity

```
df.wide$delta.oo <- df.wide$observed_otus.2 - df.wide$observed_otus.1
df.wide$delta.shannon <- df.wide$shannon.2 - df.wide$shannon.1
df.wide$delta.pd <- df.wide$PD_whole_tree.2 - df.wide$PD_whole_tree.1
```

CD4 and Viral Load

```
df.wide$delta.cd4 <- df.wide$cd4_value.2 - df.wide$cd4_value.1
df.wide$delta.vl <- df.wide$viral_load_value.2 - df.wide$viral_load_value.1
```

Subsetting the data into just the negative and the untreated to treated groups and 6-14 month sample window

```
df.wide.timefilt <- subset(df.wide, timedif.months <= 14 & timedif.months >=
    6 & group %in% c("neg", "UT"))
df.wide.timefilt.UT <- subset(df.wide.timefilt, group == "UT")
df.wide.timefilt.neg <- subset(df.wide.timefilt, group == "neg")
```

The HIV-positive cohort had a significant increase in CD4+ T cell count and decrease in viral load post-treatment with plasma HIV RNA counts between 0 and 40

```
wilcox.test(df.wide.timefilt.UT$cd4_value.1, df.wide.timefilt.UT$cd4_value.2,
    paired = T)
```

```
##
##  Wilcoxon signed rank test with continuity correction
##
## data:  df.wide.timefilt.UT$cd4_value.1 and df.wide.timefilt.UT$cd4_value.2
## V = 24, p-value = 0.01382
## alternative hypothesis: true location shift is not equal to 0
```

```
wilcox.test(df.wide.timefilt.UT$viral_load_value.1, df.wide.timefilt.UT$viral_load_value.2,
    paired = T)
```

```
##
##  Wilcoxon signed rank test
##
## data:  df.wide.timefilt.UT$viral_load_value.1 and df.wide.timefilt.UT$viral_load_value.2
## V = 136, p-value = 3.052e-05
## alternative hypothesis: true location shift is not equal to 0
```

HIV-positive individuals had significantly higher weighted but not unweighted beta diversity across time points compared to the seronegative controls, suggesting ART initiation results in significant alteration of the community composition

```
kruskal.test(uwu_distance ~ group, data = df.wide.timefilt)
```

```
##
##  Kruskal-Wallis rank sum test
##
## data:  uwu_distance by group
## Kruskal-Wallis chi-squared = 2.564, df = 1, p-value = 0.1093
```

```
kruskal.test(wu_distance ~ group, data = df.wide.timefilt)
```

```
##
##  Kruskal-Wallis rank sum test
##
## data:  wu_distance by group
## Kruskal-Wallis chi-squared = 3.8422, df = 1, p-value = 0.04998
```

There was no significant difference in the change in alpha diversity between the two time points when comparing HIV-positive individuals and seronegative controls

```
kruskal.test(delta.oo ~ group, data = df.wide.timefilt)
```

```
##
##  Kruskal-Wallis rank sum test
##
## data:  delta.oo by group
## Kruskal-Wallis chi-squared = 2.7452, df = 1, p-value = 0.09755
```

```
kruskal.test(delta.shannon ~ group, data = df.wide.timefilt)
```

```
##
##  Kruskal-Wallis rank sum test
##
## data:  delta.shannon by group
## Kruskal-Wallis chi-squared = 1.2812, df = 1, p-value = 0.2577
```

```
kruskal.test(delta.pd ~ group, data = df.wide.timefilt)
```

```
##
##  Kruskal-Wallis rank sum test
##
## data:  delta.pd by group
## Kruskal-Wallis chi-squared = 0.93369, df = 1, p-value = 0.3339
```

There was no significant association between change in alpha or beta diversity and antibiotic usage in the 6 months prior to the second sample collection Files

```
df.wide.timefilt$abx.ever <- with(df.wide.timefilt, ifelse(any(df.wide.timefilt$antibiotics_past_6_montl
    "Yes", df.wide.timefilt$antibiotics_past_6_months.2 == "Yes"),
    "Yes", "No"))
```

Beta diversity

```
kruskal.test(uwu_distance ~ abx.ever, data = df.wide.timefilt)
kruskal.test(wu_distance ~ abx.ever, data = df.wide.timefilt)
```

Alpha diversity

```
kruskal.test(delta.oo ~ abx.ever, data = df.wide.timefilt)
kruskal.test(delta.shannon ~ abx.ever, data = df.wide.timefilt)
kruskal.test(delta.pd ~ abx.ever, data = df.wide.timefilt)
```
